# A host gene expression approach for identifying triggers of asthma exacerbations

**DOI:** 10.1371/journal.pone.0214871

**Published:** 2019-04-08

**Authors:** Emily C. Lydon, Charles Bullard, Mert Aydin, Olga M. Better, Anna Mazur, Bradly P. Nicholson, Emily R. Ko, Micah T. McClain, Geoffrey S. Ginsburg, Chris W. Woods, Thomas W. Burke, Ricardo Henao, Ephraim L. Tsalik

**Affiliations:** 1 Duke University School of Medicine, Durham, NC, United States of America; 2 Duke University Center for Applied Genomics and Precision Medicine, Durham, NC, United States of America; 3 Durham Veterans Affairs Health Care System, Durham, NC, United States of America; 4 Duke University Department of Biostatistics and Informatics, Durham, NC, United States of America; Imperial College London, UNITED KINGDOM

## Abstract

**Rationale:**

Asthma exacerbations often occur due to infectious triggers, but determining whether infection is present and whether it is bacterial or viral remains clinically challenging. A diagnostic strategy that clarifies these uncertainties could enable personalized asthma treatment and mitigate antibiotic overuse.

**Objectives:**

To explore the performance of validated peripheral blood gene expression signatures in discriminating bacterial, viral, and noninfectious triggers in subjects with asthma exacerbations.

**Methods:**

Subjects with suspected asthma exacerbations of various etiologies were retrospectively selected for peripheral blood gene expression analysis from a pool of subjects previously enrolled in emergency departments with acute respiratory illness. RT-PCR quantified 87 gene targets, selected from microarray-based studies, followed by logistic regression modeling to define bacterial, viral, or noninfectious class. The model-predicted class was compared to clinical adjudication and procalcitonin.

**Results:**

Of 46 subjects enrolled, 7 were clinically adjudicated as bacterial, 18 as viral, and 21 as noninfectious. Model prediction was congruent with clinical adjudication in 15/18 viral and 13/21 noninfectious cases, but only 1/7 bacterial cases. None of the adjudicated bacterial cases had confirmatory microbiology; the precise etiology in this group was uncertain. Procalcitonin classified only one subject in the cohort as bacterial. 47.8% of subjects received antibiotics.

**Conclusions:**

Our model classified asthma exacerbations by the underlying bacterial, viral, and noninfectious host response. Compared to clinical adjudication, the majority of discordances occurred in the bacterial group, due to either imperfect adjudication or model misclassification. Bacterial infection was identified infrequently by all classification schemes, but nearly half of subjects were prescribed antibiotics. A gene expression-based approach may offer useful diagnostic information in this population and guide appropriate antibiotic use.

## Introduction

Asthma exacerbations are responsible for an estimated 1.7 million emergency department visits annually in the United States[1]. Exacerbations are frequently due to extrinsic causes such as bacterial infections, viral infections, or noninfectious causes, such as medication noncompliance or environmental exposure[2, 3]. Antibiotics are not standard of care for asthma exacerbations, and several trials have shown no benefit to the use of macrolides for asthma exacerbations[4–7]. Despite this, 22% of patients presenting to the emergency room and 58% of patients hospitalized for asthma receive antibiotics, leading to antibiotic resistance, antibiotic-related adverse events, and additional costs[8, 9]. Antibacterials and antivirals are only effective in those with confirmed bacterial and influenza infections, respectively, but it is often difficult to identify these etiologies at the point of care[10, 11].

A diagnostic test capable of identifying the cause of asthma exacerbation could help clinicians optimize treatment including antibiotic therapy. Traditional pathogen-based approaches have limitations, including sampling bias, time-to-result, and inability to distinguish infection from colonization. Consequently, host response-based diagnostics are an attractive alternative. Procalcitonin, a biomarker associated with bacterial infection, has already been utilized to guide antibiotic use in patients with asthma exacerbations, with mixed results[12–15].

We recently published microarray-derived gene expression signatures from peripheral blood that successfully distinguished bacterial, viral, and noninfectious causes of respiratory illness with 87% overall accuracy and external validation AUCs ranging from 0.90–0.99[16]. Here, we present evidence that these signatures, when translated onto a qPCR platform and tested in subjects presenting with asthma exacerbation, may differentiate between various asthma triggers. Such an approach could personalize acute care for asthmatics and mitigate emerging antibiotic resistance.

## Materials and methods

### Study design

Studies were approved by the Institutional Review Boards at Duke, University of North Carolina, Henry Ford, and Durham VA Medical Center in accordance with institutional and federal regulations regarding human subjects’ protection. Written informed consent was obtained from all subjects or legally authorized representatives.

Subjects were enrolled in the emergency departments of Duke University Medical Center, the Durham VA Medical Center, Henry Ford Hospital, and University of North Carolina Medical Center, as part of the CAPSOD (Community-Acquired Pneumonia and Sepsis Outcome Diagnostics) study (ClinicalTrials.gov NCT00258869) or as part of CAPSS (Community-Acquired Pneumonia and Sepsis Study). The objective of CAPSOD and CAPSS was to identify subjects with suspected sepsis and to collect clinical information and samples for future use. Patients were eligible for CAPSOD and CAPSS if they had a suspected acute respiratory illness and met at least two SIRS criteria. Subjects were also enrolled at Duke and the Durham VA Health Care System as part of RADICAL (Rapid Diagnostics in Categorizing Acute Lung Infection). RADICAL enrolled subjects with acute respiratory illness of suspected bacterial, viral, or noninfectious etiology. The objective of RADICAL was to develop a host response-based diagnostic assay to discriminate bacterial from viral infection.

On enrollment, subject medical history, clinical data (including vital signs, laboratory values, and radiography results), and patient-reported symptom scores were recorded. Symptoms assessed included nasal discharge, nasal congestion, sneezing, cough, malaise, throat discomfort, fever/chills, and headache. Scoring was based on the Jackson score using a zero to four scale (0: absent, 1: mild, 2: moderate, 3: severe, 4: very severe)[17–19]. Subjects also reported sick contacts and duration of illness.

Retrospective adjudications were conducted by emergency medicine, hospital medicine, pulmonary medicine, or infectious disease physicians, as previously described[16, 20]. Adjudicators had full access to the subject’s medical record, including patient history, reported symptoms, physical examination findings, clinical laboratory testing, and radiographic findings. In the absence of supporting microbiologial evidence, adjudicators could still make a classification of “suspected bacterial” or “suspected viral” infection if the clinical presentation was consistent with such an etiology. Each case was independently reviewed by two physicians. When there was disagreement, a consensus panel was convened to render a final determination. Viral PCR testing supplemented clinical microbiologic testing for all subjects; several platforms were used including ResPlex version 2.0 viral PCR multiplex assay (Qiagen), xTAG RVP FAST version 2 (Luminex), and NxTAG Respiratory Pathogen Panel (Luminex). Viral PCR testing results were provided to adjudicators but were not available at the time of treatment.

**Fig 1:**
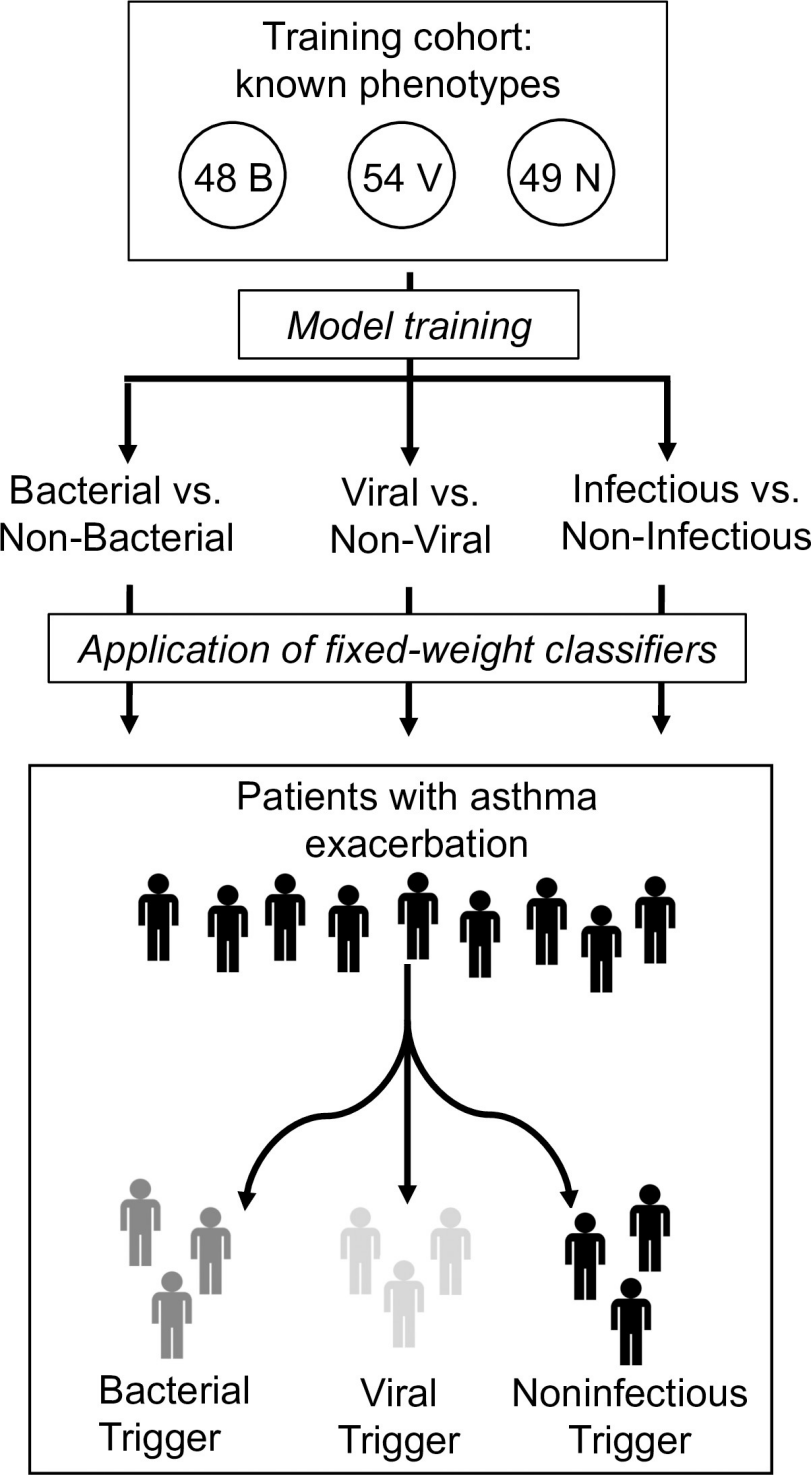
Overview schematic. Gene expression data from 48 bacterial (B), 54 viral (V), and 49 noninfectious (N) microbiologically confirmed cases were used to train classifiers capable of distinguishing between bacterial, viral, and noninfectious causes of illness. Specifically, the model created 3 separate classifiers: bacterial versus non-bacterial, viral versus non-viral, and infectious versus non-infectious. The fixed-weight classifiers were then applied to a cohort of patients with acute asthma exacerbations of various etiologies to predict the underlying trigger of the exacerbation.

In order to apply the gene expression-based classification model, that model must first be trained on a cohort of known phenotype. The training cohort included 151 subjects who presented with respiratory tract illness and had clinically adjudicated and microbiologically confirmed bacterial, viral, or noninfectious illness. The validation cohort included 46 subjects with asthma exacerbation adjudicated as having bacterial, viral, or noninfectious triggers (Fig 1). Both the training cohort and validation cohort were retrospectively selected from the same pool of subjects enrolled through CAPSOD, CAPSS, and RADICAL. In the validation cohort, presence of asthma exacerbation was determined by the adjudicators on the basis of a pre-existing diagnosis of asthma in the electronic medical record, physical examination findings of wheezing and shortness of breath, and a compatible treatment plan including beta agonists and steroids. Information about antibiotic and oseltamivir prescription were collected.

### Clinical data analysis

Patient-reported symptom scores, illness duration, presence of sick contacts, and clinical findings were analyzed to determine statistically significant differences between bacterial, viral, and noninfectious cases. Clinical findings assessed included those routinely available at the time of clinical evaluation: vital signs, white blood cell (WBC) count, and radiography. Scores for sneezing, nasal discharge, and nasal congestion were averaged to obtain a composite of nasal symptoms. Similarly, all symptom scores for each patient were summed to achieve a composite representing overall symptom severity. Continuous variables were analyzed using the Kruskal-Wallis statistical test, and binary variables were analyzed with the Fisher’s exact statistical test[21, 22]. Statistical tests were employed using R version 3.5.0[23].

### Procalcitonin measurement

Procalcitonin was measured for all subjects in the asthma exacerbation cohort. Serum samples were measured on the Roche Elecsys 2010 analyzer (Roche Diagnostics) or miniVIDAS immunoassay (bioMérieux). Plasma samples were measured using B·R·A·H·M·S Procalcitonin sensitive KRYPTOR (Thermo Fischer Scientific). Values >0.25 μg/liter assigned patients as having a bacterial trigger for asthma, while values ≤0.25 μg/liter were considered non-bacterial[24].

### Gene expression

At initial presentation, peripheral whole blood was collected from each subject. Total RNA was extracted using PAXgene Blood miRNA kit (Qiagen) according to the manufacturer’s protocol. RNA quantity and quality were assessed by NanoDrop Spectrophotometer (Thermo Scientific) and Aligent 2100 Bioanalyzer with RNA 6000 Nano Chip, respectively. A complementary DNA library was generated from total RNA using SuperScript VILO MasterMix (Thermo Scientific). Semi-quantitative, real-time PCR was performed on TaqMan Low Density Arrays (TLDA). Although TLDA cards can accommodate 384 targets, these were customized to quantify 87 gene targets, which was the number that maximized performance accuracy. A complete list of gene targets is included in S1 Table. These targets were selected from prior microarray-based studies in an iterative process, substituting poorly performing assays with different probes for the same transcript or with other transcripts that were highly correlated with the original[16, 25].

### Model training and validation

Due to technical differences between microarray and qPCR, it was necessary to derive a new prediction model on qPCR data from a new training cohort. First, raw gene expression results were averaged normalized against two reference probes. Exploratory data analysis revealed differences between the two sample batches, which were corrected using an empirical Bayesian frameworks model[26, 27]. The normalized, batch-corrected data was used to fit a logistic regression model to perform both classification and target weight selection using the R package “glmnet”[28]. Specifically, we used Least Absolute Shrinkage and Selection Operator for regularization and performed nested cross validation to select parameters. This resulted in three independent binary classifiers (bacterial versus non-bacterial, viral versus non-viral, and noninfectious versus infectious), which were combined into a single decision model using a one-versus-all scheme where the largest probability determines class membership. The fixed-weight model was then applied to the asthma exacerbation validation cohort to determine host-response classification. Since the asthma validation cohort was measured in an independent TLDA experiment, we included technical replicates in both training and validation TLDA experiments to evaluate and potentially correct for batch differences. In addition to this supervised analysis, we also performed dimensionality reduction on the asthmatic cohort to visualize the clustering of different groups based on infection status with respect to all 87 targets. The specific clustering algorithm utilized was T-distributed stochastic neighbor with a perplexity parameter of 10, which was implemented with the R package “Rtsne”[29]. All scripts were written in R version 3.5.0[23].

## Results

### Clinical cohorts

The training cohort included 151 subjects with acute respiratory illness of bacterial (n = 48), viral (n = 54), or noninfectious etiology (n = 49) (Table 1). Noninfectious illness was included instead of healthy controls because they represent a more clinically relevant population for diagnostic testing. Noninfectious etiologies are also a frequent cause of asthma exacerbation, further justifying their inclusion. Ethnicity and gender were well-balanced across groups. The viral group exhibited lower illness severity, as inferred from hospital admission rates (39% for viral vs. 100% for bacterial and 48% for noninfectious), and were younger (mean 42 years) compared to the bacterial (54 years) and noninfectious groups (58 years).

**Table 1:** Demographic information for the training and asthma exacerbation validation cohorts.

Category	*Training Cohort*				*Asthma Validation Cohort*			
	Bacterial (n = 48)	Viral (n = 54)	Noninfectious (n = 49)	Total (n = 151)	Bacterial (n = 7)	Viral (n = 18)	Noninfectious (n = 21)	Total (n = 46)
Males, n (%)	24 (50%)	20 (37%)	32 (65%)	76 (50%)	2 (29%)	5 (28%)	10 (48%)	17 (37%)
Age, mean (range)	54 (16–88)	42 (14–88)	58 (21–87)	51 (14–88)	38 (6–70)	40 (19–73)	34 (8–55)	37 (6–73)
Race, n (%)								
*Black*	24 (50%)	28 (52%)	21 (43%)	73 (48%)	3 (43%)	12 (67%)	18 (86%)	33 (72%)
*White*	23 (48%)	23 (43%)	27 (55%)	73 (48%)	4 (57%)	5 (28%)	1 (5%)	10 (22%)
Other*	1 (2%)	3 (6%)	1 (2%)	5 (3%)	0 (0%)	1 (6%)	2 (10%)	3 (7%)
Admitted, n (%)	46 (96%)	16 (30%)	42 (86%)	104 (69%)	7 (100%)	7 (39%)	10 (48%)	24 (52%)

* Other includes Asians, American Indians/Alaska Natives, Native Hawaiians/Pacific Islanders, and those who chose not to report their race.

An independent validation cohort of 46 subjects with asthma exacerbation was identified (Table 1). Of these, 7 subjects (15%) were clinically adjudicated as having a bacterial infection, though none had confirmatory microbiology. Eighteen subjects (39%) had a viral infection, with a viral etiology identified in all cases. Influenza and rhinovirus were most frequently identified (7 cases each). Twenty-one subjects (46%) were adjudicated as having a noninfectious asthma exacerbation etiology including medication noncompliance, seasonal allergies, smoking/illicit drug use, and dust exposure. The asthma validation group had more females and was younger than the training cohort, reflective of typical asthma demographics[30]. Race and severity of illness were similar across cohorts.

We first looked at readily available clinical data to understand the grounds for clinical adjudication and to determine whether clinical variables alone could distinguish between groups (Table 2). Pulse oximetry was lower in the bacterial group (mean 91% vs. 97% for viral and 97% for noninfectious, p = 0.006), but temperature was similar. A complete blood count (CBC) was more frequently obtained in the bacterial cohort (in 100%, 61%, and 43% of cases, respectively, p = 0.02), and, of those tested, mean WBC count was higher in the bacterial group (14.0x10^9^/L, 9.4x10^9^/L, and 9.8x10^9^/L, respectively, p = 0.04). WBC differential, however, was similar. Imaging tended to be ordered more frequently in the bacterial group. Of those with imaging studies, radiographic abnormalities were more common in the bacterial group compared to the viral and noninfectious groups (100%, 20%, and 38%, respectively; p<0.001). Radiographic abnormalities included bilateral opacities, unilateral opacities, bronchial thickening, reticular markings, adenopathy, and nodules.

**Table 2:** Clinical data from asthma exacerbation cohorts.

Clinical Adjudication	Temperature (°C) Mean (range)	O_2_ Saturation (%) Mean (range)	WBC (x10^9^/L) Mean (range)*	Radiographic abnormalities^+^
Bacterial (n = 7)	36.8 (35.8–38.2)	91 (86–97)	14 (5.9–21.7)	100%
Viral (n = 18)	37.2 (35.6–38.8)	96.8 (89–100)	9.4 (5.9–15.9)	20%
Noninfectious (n = 21)	36.8 (36.0–37.8)	96.6 (91–100)	9.8 (5.8–14.3)	38%

*CBC was not obtained in all individuals. 100% of bacterial cohort, 61% of viral cohort, and 43% of noninfectious cohort had a CBC ordered on admission.

^+^Radiography was not obtained in all individuals. 100% of the bacterial cohort, 83% of the viral cohort, and 62% of the noninfectious cohort had radiography (either chest x-ray or CT scan) ordered on admission.

**Fig 2:**
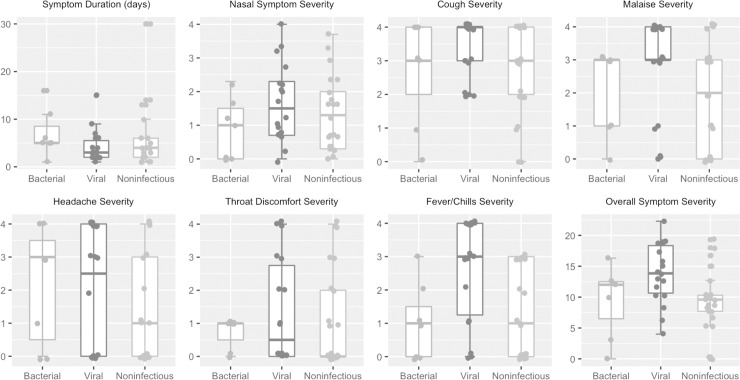
Symptom scores across clinically adjudicated phenotypes. Illness duration and symptom scores from each subject were compared between the different clinically adjudicated phenotypes. Symptom scores were graded on a 0 to 4 scale, with 0 indicating not present and 4 indicating “very severe”. Nasal symptoms represented an average of nasal discharge, nasal stuffiness, and sneezing. Overall symptom severity was simply the sum of all symptom scores. Most symptoms were similar between groups. Only two reached statistical significance: fever/chills (p = 0.005) and a composite of overall symptom severity (p = 0.02). Kruskal-Wallis statistical test was used to assess significance.

Next, we determined whether patient-reported symptom scores correlated with adjudication (Fig 2). Most clinical symptoms were similar between groups. Duration of illness and presence of sick contacts were also similar. Only fever/chills (p = 0.006) and a composite of all symptom scores (p = 0.02) were higher in viral infection cases than bacterial and noninfectious cases. No parameters distinguished bacterial and noninfectious groups.

### Host gene expression-based classification

A model was derived on the training cohort described above, yielding three independent classifiers: bacterial versus non-bacterial, viral versus non-viral, and noninfectious versus infectious (Fig 1). Leave-one-out cross validation within the training cohort revealed accurate discrimination between bacterial [area under the receiving operator curve, (AUC) 0.85], viral (AUC 0.89), and noninfectious illness (AUC 0.88). The model created in this training cohort was fixed and applied to 46 cases of asthma exacerbation.

Technical differences and batch effects between the training and asthma experiments could obscure the results. This was mitigated by the use of internal normalization standards and technical replicates across experiments. These strategies resulted in excellent correlation between experiments (R^2^ = 0.90), indicating the RT-PCR data from the training and validation cohorts could be directly compared using a fixed-weight model without the need for batch correction.

**Fig 3:**
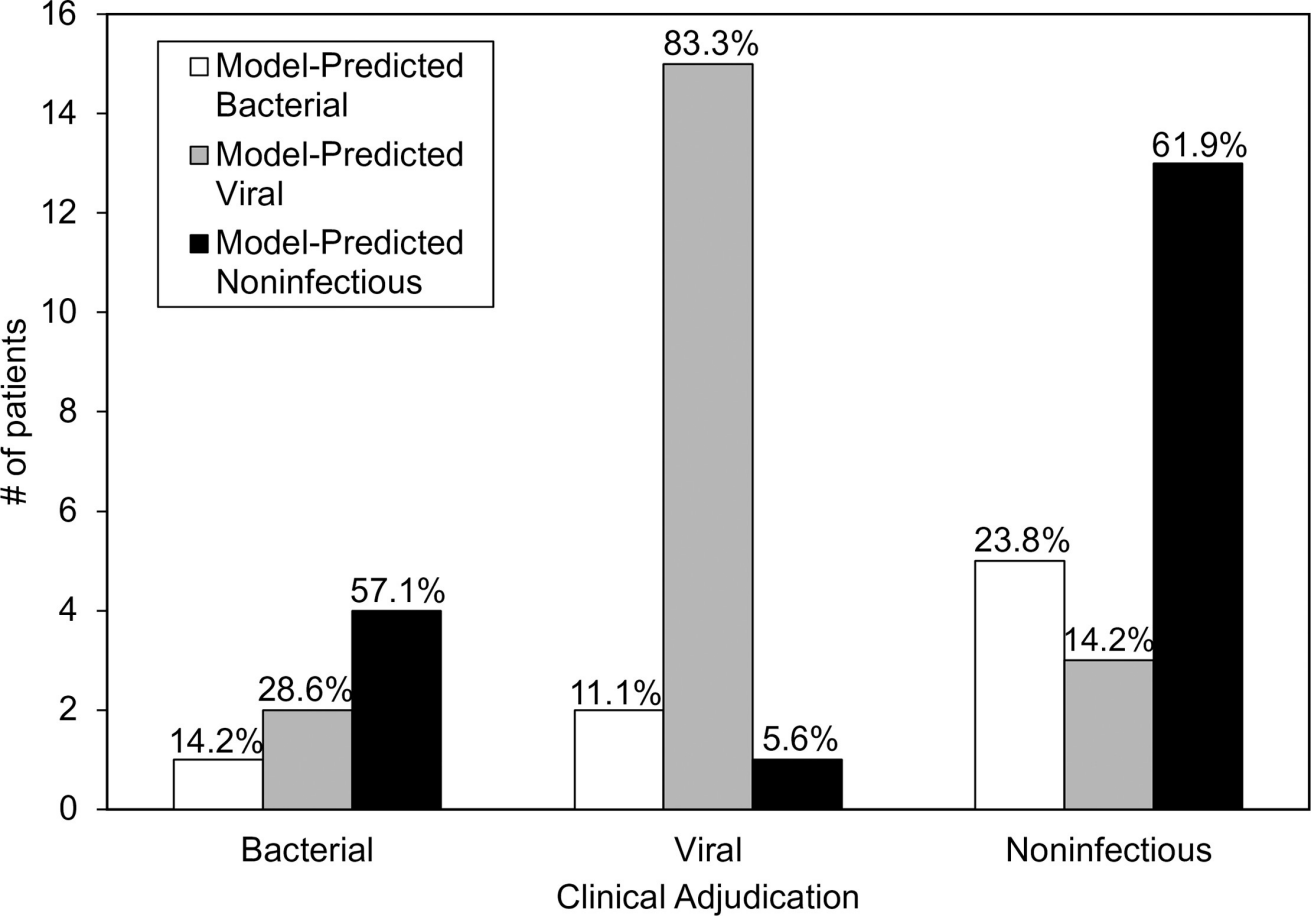
Gene expression classification of the asthma exacerbation cohort. The fixed-weight gene expression model developed in the training cohort was applied to each of the 46 asthma exacerbation cases to determine gene expression-based classification. Clinical adjudication groups were subsequently stratified by gene expression class, with the respective percentages indicated at the top of each bar. Viral and noninfectious groups had a high degree of agreement between adjudication and model classification (83.3% and 61.9%, respectively), but those adjudicated bacterial were often classified as viral or noninfectious by the model.

Applying the fixed-weight model developed in the training cohort, we assigned a class (bacterial, viral, or noninfectious) to each of the 46 asthma cases (Fig 3). The model identified eight (17%) bacterial infections, twenty (43%) viral infections, and eighteen (39%) noninfectious cases. The proportions were similar to the clinically adjudicated groups, supporting the fact that viral and noninfectious triggers cause the majority of asthma exacerbations. However, there were some differences between the gene expression assignments and clinical adjudications. In the subset of asthma exacerbations clinically adjudicated as bacterial, only one had a gene expression signature consistent with bacterial infection; instead, 2 (29%) were classified as viral, and 4 (57%) were noninfectious based on gene expression. Among the cases adjudicated as viral, all of whom had an identified viral etiology, 83.3% were identified as viral by gene expression. Within the noninfectious cohort, there was 61.9% agreement between adjudication and gene expression; whereas gene expression identified 23.8% as bacterial and 14.3% as viral.

**Fig 4:**
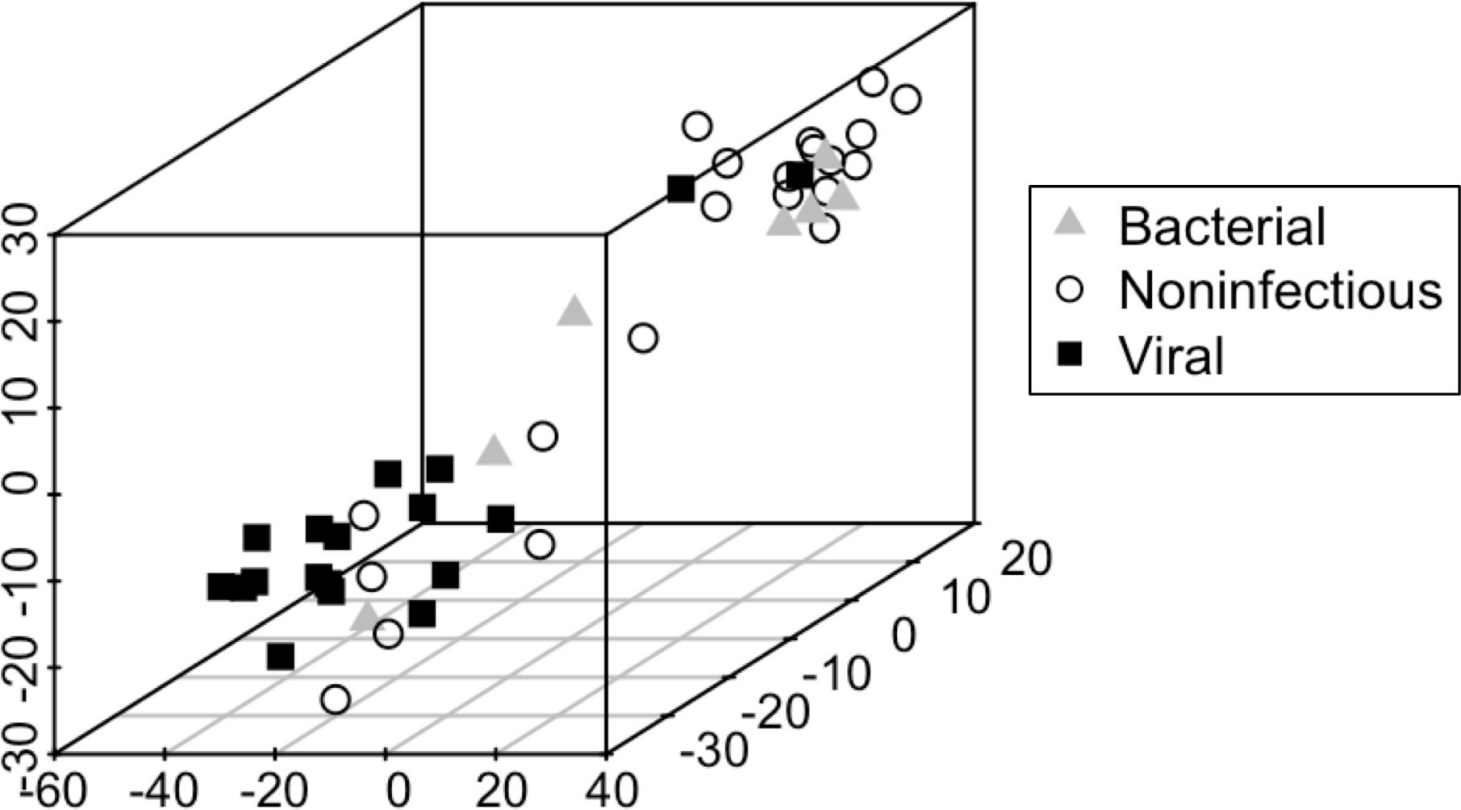
Dimensionality reduction to visualize differences between asthma exacerbation subgroups with respect to all gene targets. Unsupervised analysis using t-distributed stochastic neighbor embedding (TSNE) was utilized to depict the visual relationships between all 46 subjects with asthma exacerbations. Each subject is labeled by their respective bacterial, viral, or noninfectious status, based on clinical adjudication. Notably, in TSNE, the axes are not meant to be interpretable; this plot represents a 3D projection of higher dimensional space.

In addition to applying the fixed weight model, we explored whether the 87 gene targets could independently cluster subjects by infection status using unsupervised dimensionality reduction to display the “closeness” of each group (Fig 4). Consistent with earlier results, the viral and noninfectious groups separated well. Bacterial cases did not form their own group but clustered either with viral or non-infectious patients.

Given the discrepancies between adjudication and gene expression for the bacterial cases, we explored the clinical features of these discrepant cases. Two adjudicated bacterial cases were classified as viral by the model. One had patchy airspace consolidation on x-ray, and the other had a clear x-ray on enrollment but lobar infiltrates on repeat imaging two days later. Four subjects were adjudicated as bacterial and classified as noninfectious by the model. All had radiographic abnormalities on x-ray, described as “biapical opacities”, “atelectasis versus pneumonitis”, “multilobar consolidation”, and “lobar infiltrate”. All four subjects also had leukocytosis, but only one had an elevated neutrophil percentage and none had bandemia. Only one was febrile.

### Procalcitonin

Procalcitonin was measured for all 46 subjects in the asthma cohort. Notably, procalcitonin was not available to adjudicators when assigning clinical categories enabling its use as an independent comparator. Using 0.25 μg/liter as the threshold, procalcitonin classified only one subject as bacterial, further evidence that bacterial infections are infrequent in this population. Interestingly, this particular subject was clinically adjudicated as viral and tested positive for rhinovirus, but consistent with the procalcitonin results, was predicted bacterial by gene expression.

### Antibiotic usage

**Fig 5:**
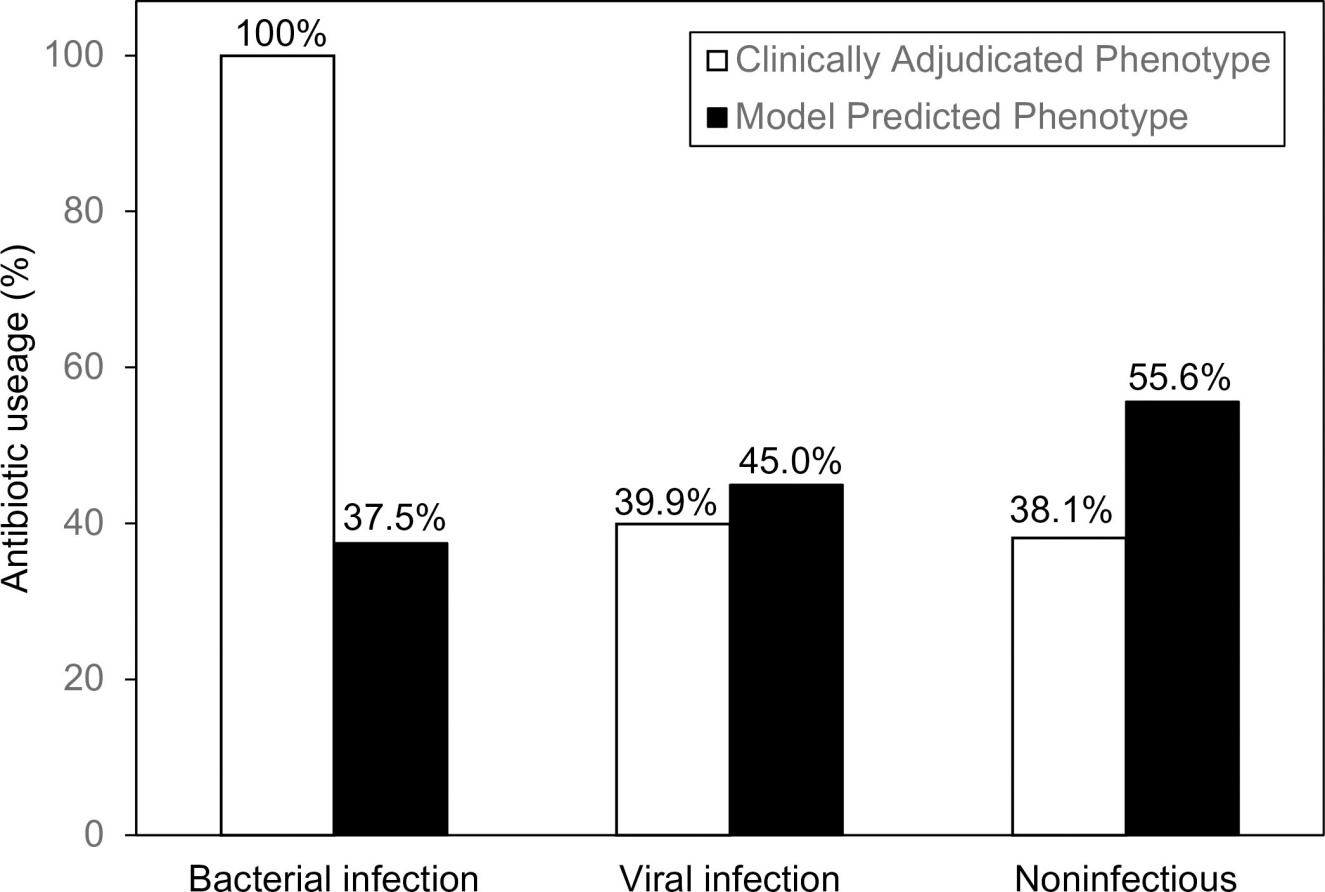
Frequency of antibiotic prescription in different infection classes. Antibiotic prescription during emergency room or hospital stay was recorded for each subject. Antibiotic prescription rate was calculated for the clinical adjudication groups and this was compared to the antibiotic prescription rate in the model-predicted groups. The respective percentages are indicated at the top of each bar. Antibiotics were prescribed more frequently for subjects with viral and noninfectious signatures than for subjects with bacterial signatures.

Antibiotics were prescribed in 47.8% of all cases, substantially higher than the percentage of either adjudicated or model-predicted bacterial infections (Fig 5). Fluoroquinolones and macrolides were the most frequently prescribed classes. When antibiotic usage was subdivided by model-predicted phenotype, antibiotics were prescribed more frequently in cases with viral and noninfectious signatures than cases with bacterial signatures, further supporting the observation that antibiotics are frequently used even when bacterial infection is unlikely by clinical or diagnostic criteria.

Oseltamivir was prescribed for 9 individuals despite only a third testing positive for influenza. The rest were negative for all viruses on clinical respiratory virus testing (n = 4), or did not undergo clinical respiratory virus testing (n = 2). The rationale for oseltamivir use in these cases was influenza-like illness, despite negative or absent clinical testing for influenza. Notably, research-based supplemental PCR testing identified coronavirus and parainfluenza in two of these subjects, but this was not known at the time of clinical treatment.

## Discussion

In this study, we applied a host response signature to subjects presenting with asthma exacerbations and classified them by their underlying bacterial, viral, or noninfectious gene expression patterns. We compared this approach to clinical adjudication and procalcitonin. Our results indicated that peripheral blood gene expression profiles may provide additional clinically useful information for distinguishing the major classes of asthma exacerbation triggers. In the acute care setting, such a task is challenging since asthma exacerbations often have similar presentations and existing diagnostics fail to identify an underlying cause in most. As a result, antibiotics are overprescribed—nearly half of the individuals in this study were given antibiotics, despite only a handful being adjudicated as having bacterial infection. This impacts antibiotic stewardship efforts as well as patient outcomes; a recent study reported longer admissions and higher cost of stay in asthmatics treated with antibiotics[31]. Gene expression approaches, once translated onto a clinically useful platform, could help abate empiric prescribing practices, mitigate antibiotic resistance, and improve patient care.

Our study is the first use of a host gene expression classifier to identify infectious and noninfectious causes of asthma exacerbation, but many other studies have utilized gene expression profiling to better understand asthma pathogenesis. Measures of the host response have been used to aid the initial diagnosis of asthma[32, 33], stratify asthma by severity and driving immune response[34–38], and identify those at risk for frequent exacerbations[39, 40]. Furthermore, other studies have characterized the molecular response of asthmatics to viral infection[41, 42]. Our work supplements the growing body of research that will hopefully lead to personalized asthma care.

One of the challenging aspects of diagnostic development is the lack of a perfect gold standard–if clinical intuition were perfect, there would be no need for additional diagnostic tests. Therefore, discordance between clinical adjudication and model classification may have arisen from errors in either classification scheme. Most of the discordant classifications involved the bacterial subgroup, for which there are several likely explanations. The seven individuals clinically identified as having bacterial infections had no supporting microbiology. Therefore, clinical adjudications were assigned based on symptoms, physical examination, laboratory testing, and radiography, all of which poorly discriminate bacterial and viral infections[43, 44]. Radiographic abnormalities, which were the most important factor for bacterial adjudication in this study, are frequently present in viral infections[45–48]. Therefore, the subjects adjudicated as bacterial but classified as viral may have been true viral infections. There are other potential reasons for the observed discordance in bacterial vs. noninfectious diagnoses. The responses to bacterial and noninfectious inflammation have a large amount of biological overlap compared to the more specific interferon signaling in viral infections[16]. Consistent with this, we observed that the majority of discordance occurred between these two groups. In this scenario, a false negative bacterial infection diagnosis is of greatest concern due to the potentially missed opportunity to treat with antibiotics. Further iterations of the model will need to address rates of false negative bacterial diagnoses and the clinical cost of such errors.

In this study, procalcitonin identified only one subject as bacterial. The prior success of procalcitonin to guide antibiotic use is tempered by recent studies raising some doubts. One large meta-analysis estimated only 77% sensitivity in sepsis while another study showed 67% sensitivity in discriminating bacterial and viral infection [14, 49]. A recent randomized controlled trial showed that procalcitonin-guided antibiotic administration did not lessen antibiotic use, due primarily to concerns that a bacterial infection was still present[15]. In contrast our gene expression-based strategy offers independent information about viral and bacterial etiologies, which may provide the necessary reassurance.

One limitation of this study is sample size. We attempted to augment the analysis using publicly available datasets, however, the available asthma exacerbation cohorts did not include bacterial, viral, and noninfectious etiologies. Due to technical differences between our RT-PCR experiments and those in the public domain (microarray or sequencing), our fixed weight model could not be applied; a new model would need to be trained. In order to do so, all three phenotypes must be present. Future directions include the development of an asthma exacerbation-specific signature in a larger, prospectively enrolled cohort, which could also incorporate clinical variables and procalcitonin into the model. Ideally, such a cohort would have supporting microbiological evidence to provide higher confidence in clinical adjudication and allow calculation of performance characteristics. Additionally, our enrollments focused on patients with acute respiratory illness or suspected sepsis. They did not focus exclusively on patients with asthma, which may have resulted in some bias compared to a dedicated, prospective asthma study. Additionally, there is some uncertainty in a pre-existing diagnosis of asthma in the electronic medical record, a common dilemma facing emergency medicine clinicians. Another limitation was the age of our cohort. Asthma exacerbations often occur in younger individuals, but our cohort was largely adult (though several pediatric subjects were included). However, the signatures used for this study were previously evaluated in both adult and pediatric patients, showing no significant difference based on age[16]. Nevertheless, assessing performance on younger subjects with asthma, and more generally understanding the impact of age on genomic measures of the host response, is an important next step that we are currently undertaking.

In conclusion, host transcriptomic signatures may offer useful diagnostic information to aid in management of patients with asthma exacerbation. Confirmation would require a larger cohort that includes microbiologically confirmed bacterial infection or ideally, a prospective, randomized trial that uses host gene expression to guide therapy. The RT-PCR platform described in this study requires several hours of sample processing time, which is too long to meaningfully impact most clinical applications. However, further translation of this gene expression signature onto a clinically useful platform is currently underway, with current PCR-based methods yielding a time-to-result of approximately 45 minutes[50–52]. Gene expression-based approaches, procalcitonin, and clinical intuition all offer different strengths and weaknesses, and a combination of all may be the best approach to improve the management of asthma exacerbations.

## Supporting information

S1 Table Model gene targets. Probes included in the customized RT-PCR platform, selected from prior microarray-based studies, and ordered alphabetically.
(DOCX)
