# Supplementary material for: A host gene expression approach for identifying triggers of asthma exacerbations
Source: PLoS One. 2019 Apr 8;14(4):e0214871. doi: 10.1371/journal.pone.0214871 (PMC6453459; doi:10.1371/journal.pone.0214871)
Supplement: S1 Table — Probes included in the customized RT-PCR platform, selected from prior microarray-based studies, and ordered alphabetically. (DOCX) [file pone.0214871.s001.docx]

**S1 Table: Model Gene Targets.** Probes included in the customized RT-PCR platform, selected from prior microarray-based studies, and ordered alphabetically.

| Probe # | TaqMan Gene Name | TaqMan Assay ID* |
| --- | --- | --- |
| 1 | ARL1 | Hs01029870_m1 |
| 2 | ARPC3 | Hs00855185_g1 |
| 3 | ATF3 | Hs00910173_m1 |
| 4 | ATG2A | Hs00390076_m1 |
| 5 | BATF | Hs00232390_m1 |
| 6 | CADM1 | Hs00296064_s1 |
| 7 | CBX7 | Hs00545603_m1 |
| 8 | CCDC19 | Hs01099244_m1 |
| 9 | CD160 | Hs00199894_m1 |
| 10 | CD302 | Hs00208436_m1 |
| 11 | CD44 | Hs00153304_m1 |
| 12 | CD63 | Hs00156390_m1 |
| 13 | CHI3L1 | Hs01072230_g1 |
| 14 | CIB2 | Hs00197280_m1 |
| 15 | CLC | Hs01055743_m1 |
| 16 | CTBP1 | Hs00972289_g1 |
| 17 | CYP27A1 | Hs01017992_g1 |
| 18 | DDX58 | Hs01061436_m1 |
| 19 | ERC1 | Hs00327390_s1 |
| 20 | EXOG | Hs01035290_m1 |
| 21 | EXOSC4 | Hs00363401_g1 |
| 22 | FAM134C | Hs00738661_m1 |
| 23 | FNBP4 | Hs01553131_m1 |
| 24 | FOLR3 | Hs01549264_m1 |
| 25 | GGT1 | Hs00980756_m1 |
| 26 | GIT2 | Hs00331902_s1 |
| 27 | GLIPR1 | Hs00199268_m1 |
| 28 | GLUD1 | Hs03989560_s1 |
| 29 | HERC1 | Hs01032528_m1 |
| 30 | HERC5 | Hs01061821_m1 |
| 31 | DRB1 | Hs00734212_m1 |
| 32 | HNRNPA0 | Hs00246543_s1 |
| 33 | IFI27 | Hs01086373_g1 |
| 34 | IFI44 | Hs00951349_m1 |
| 35 | IFI44L | Hs00915294_g1 |
| 36 | IFI6 | Hs00242571_m1 |
| 37 | IFIT1 | Hs01911452_s1 |
| 38 | IFIT1 | Hs03027069_s1 |
| 39 | IFIT2 | Hs01922738_s1 |
| 40 | IFIT3 | Hs01922752_s1 |
| 41 | IFITM1 | Hs00705137_s1 |
| 42 | IFNGR2 | Hs00985251_m1 |
| 43 | IL1RN | Hs00893626_m1 |
| 44 | IRF2 | Hs01082884_m1 |
| 45 | ISG15 | Hs01921425_s1 |
| 46 | ITPR3 | Hs00609948_m1 |
| 47 | KIAA1324 | Hs00381767_m1 |
| 48 | KIDINS220 | Hs01057000_m1 |
| 49 | LAMP3 | Hs00180880_m1 |
| 50 | LAPTM4B | Hs00363282_m1 |
| 51 | LY6E | Hs03045111_g1 |
| 52 | MCTP1 | Hs01115711_m1 |
| 53 | MX1 | Hs00182073_m1 |
| 54 | OAS1 | Hs00973637_m1 |
| 55 | OAS2 | Hs00213443_m1 |
| 56 | OAS3 | Hs00934282_g1 |
| 57 | OASL | Hs00984390_m1 |
| 58 | ORAI2 | Hs00259863_m1 |
| 59 | PDE3B | Hs01057217_m1 |
| 60 | PPCDC | Hs00222418_m1 |
| 61 | PRF1 | Hs00169473_m1 |
| 62 | RAB7L1 | Hs00187510_m1 |
| 63 | RABGAP1L | Hs02567906_s1 |
| 64 | RPS21 | Hs00963477_g1 |
| 65 | RSAD2 | Hs00369813_m1 |
| 66 | RTCB | Hs00204783_m1 |
| 67 | RUNX1 | Hs00231079_m1 |
| 68 | SEPT4 | Hs00910209_g1 |
| 69 | SERPING1 | Hs00934330_m1 |
| 70 | SIGLEC1 | Hs00988063_m1 |
| 71 | SMPD1 | Hs01086851_m1 |
| 72 | SP100 | Hs00162109_m1 |
| 73 | SPATS2L | Hs01016364_m1 |
| 74 | SPI1 | Hs00231368_m1 |
| 75 | STAP1 | Hs01038134_m1 |
| 76 | TAF4 | Hs01122669_m1 |
| 77 | TGIF1 | Hs00820148_g1 |
| 78 | TMEM165 | Hs00218461_m1 |
| 79 | TNFAIP6 | Hs01113602_m1 |
| 80 | TNFSF10 | Hs00234356_m1 |
| 81 | TRMT13 | Hs00219487_m1 |
| 82 | USP18 | Hs00276441_m1 |
| 83 | ZER1 | Hs01115240_m1 |

*Additional information regarding assay IDs is available at https://www.thermofisher.com/order/genome-database/details/gene-expression/.
